# Drilling around the corner: a comprehensive literature review of steerable bone drills

**DOI:** 10.3389/fmedt.2025.1426858

**Published:** 2025-04-09

**Authors:** Esther P. de Kater, Paul Breedveld, Aimée Sakes

**Affiliations:** Department of BioMechanical Engineering, Faculty of Mechanical Engineering, Delft University of Technology, Delft, Netherlands

**Keywords:** bone drilling, design, mechanical design, orthopedics, steerable drilling

## Abstract

**Introduction:**

Orthopedic procedures often require drilling of tunnels through bone, for instance for the introduction of implants. The currently used rigid bone drills make it challenging to reach all target areas without damaging surrounding anatomy. Steerable bone drills are a promising solution as they enable access to larger volumes and the creation of curved tunnels thereby reducing the risk of harm to surrounding anatomical structures.

**Method:**

This review provides a comprehensive overview of steerable bone drill designs identified in patent literature via the Espacenet database and in scientific literature accessed via the Scopus data base. A Boolean search combined with pre-set inclusion criteria returned 78 literature references describing a variety of drill designs.

**Results:**

These drill designs could be categorized based on how the drilling trajectory was defined. Three methods to influence the drilling trajectory were identified: (1) the device (57% of the sources), (2) the environment (15% of the sources): the path is defined based on the tissue interaction forces with the surrounding bone or (3) the user defines the drilling trajectory (28% of the sources).

**Discussion:**

The comprehensive overview of steerable drilling methods provides insights in the possibilities in drill design and may be used as a source of inspiration for the design of novel steerable drill designs.

## 1 Introduction

### 1.1 Steerable bone drilling

**Figure 1:**
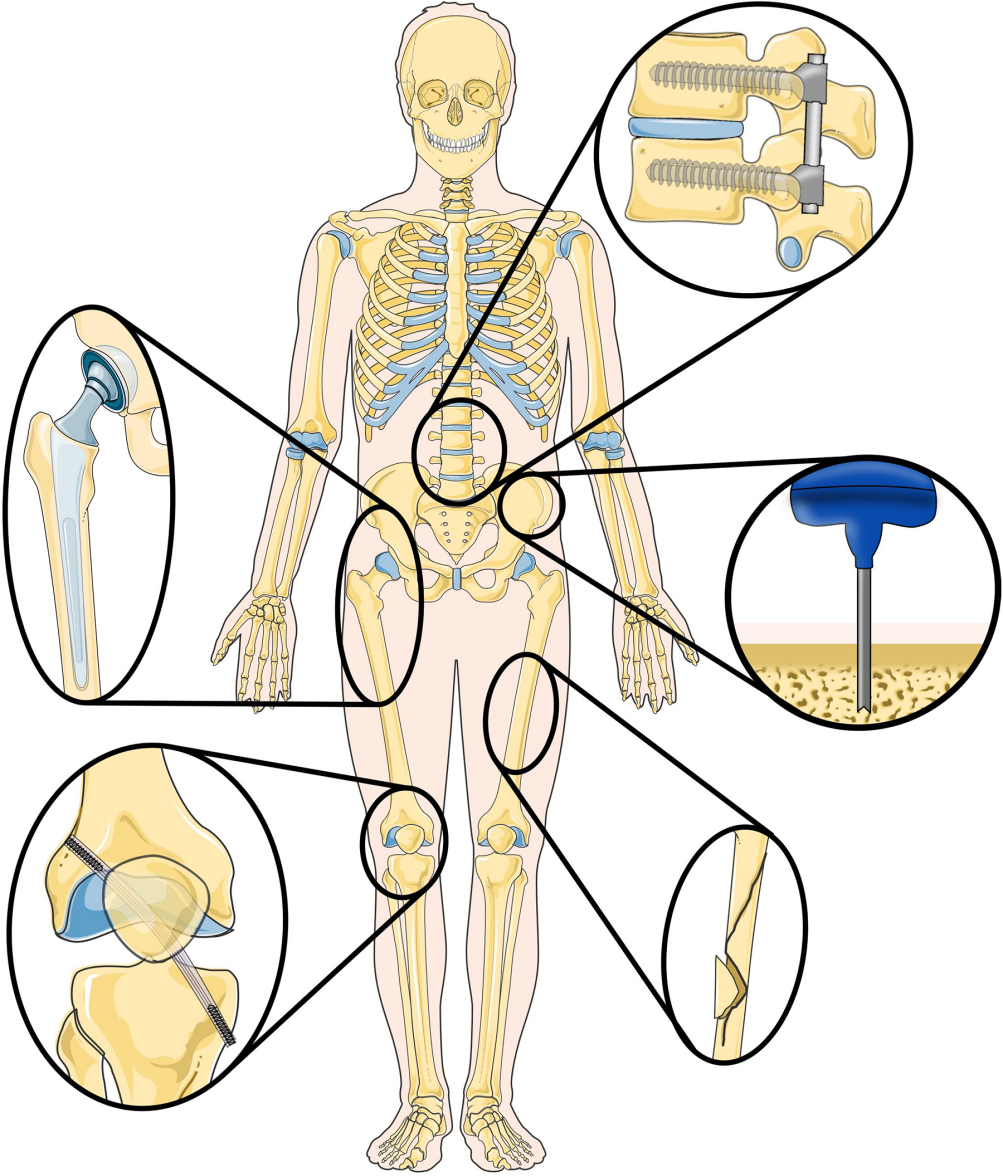
Overview of (orthopedic) procedures where the use of steerable bone drills can provide advantages, including implant placement, ligament reconstruction, fracture fixation and bone harvesting. Illustration adapted from Servier Medical Art by Servier (https://smart.servier.com/), licensed under a Creative Commons Attribution 3.0 Unported License.

Bone drilling is widely employed surgical technique in various medical procedures, playing an important role in fracture fixation, implant placement, and facilitating access to specific treatment sites (Figure 1) (1). Bone drills are usually rigid devices containing an axially rotating cutting head. They are often used in conjunction with surgical screws and plates to treat a wide variety of injuries and diseases. Although bone drills are effective in open surgery, the strive for minimal invasive surgeries and optimal patient outcomes poses a challenge when using conventional rigid and straight bone drills especially, when navigating through tight spaces, such as joints (2).

For instance, Anterior Cruciate Ligament (ACL) reconstruction aims to restore knee function and eliminate pain and discomfort caused by a damaged or torn ACL (3). Precision drilling is crucial to create the tunnels in the femur and tibia such that the damaged or torn ligament can be reattached with a pin (4). A reconstruction of the ACL that more closely resembles the anatomical structure before injury is associated with an improved biomechanical outcome (5). However, drilling of the tunnels required for this anatomical reconstruction with rigid drills is challenging (6). Similarly, osteonecrosis treatment, particularly in the femoral head, involves drilling to remove lesions areas to prevent collapse of the femoral head due to disrupted blood supply (7). Conventional rigid drills complicate the removal of the entire lesion area without causing substantial damage to surrounding healthy tissue.

The integration of steerable bone drills in orthopedic procedures offers the potential to remove entire lesions through a single entry point, as the steerability allows for reaching specific locations within the bone, and drilling along preferred trajectories, thus minimizing damage to surrounding anatomy (8). Furthermore, steerable drills may enable alternative drilling trajectories to reach lesions, further minimizing damage to healthy surrounding tissue. The versatility of steerable bone drills presents a promising solution across various medical procedures, as the steerability potentially increases the precision, reduces the complication rates improves patient outcomes.

Steerable bone drills, with enhanced maneuverability, offer potential benefits across a spectrum of (orthopedic) procedures. In procedures such as bone harvesting, where complications such as pain, nerve injury, and fracture may occur (9), steerable bone drills might provide a solution to minimize these risks. Especially in patients with compromised bone quality due to osteoporosis, fractures may occur if multiple grafts are taken too close together in order to obtain sufficient graft material (10).

Despite their clear utility, only a limited number of these steerable bone drills are currently available for clinical use. Achieving a balance between the required flexibility for bending and steering while simultaneously accomplishing the required axial rigidity to facilitate bone drilling is challenging. Examples of commercially available bone drills are the *Stryker MicroFX OCD* (11) and the *Carevature Dreal* (12). These flexible drills are used in combination with a curved external guide, facilitating easier access to entry points without compromising surrounding anatomy. However, the drilling trajectory through the bone remains fixed, lacking the flexibility for clinicians to adapt the trajectory during the procedure.

### 1.2 Goal of this study

While commercially available steerable bone drills are still in their initial stages, a multitude of innovative designs are documented in scientific and patent literature. Currently available reviews on bone drilling focus on scientific literature (1, 13) describing the parameters affecting the drilling performance and thermal reactions of rigid drills, with limited attention given to the validation and practical application of steerable designs. The review of Sendrowicz *et al*. (14) focuses on steerable bone drill designs presented in patent literature, however the validation of these designs and the drilling performance of these designs is limited. The current study expands its focus by incorporating both scientific literature and patent literature, as scientific literature provides information on the current capabilities of steerable bone drills, while patent literature sheds a light on potential future development areas.

This review aims to present a comprehensive overview of steerable bone drills, highlighting their ability to create curved tunnels for the treatment of challenging lesion areas, or to drill specific paths to reach specific target locations and improve the placement of implants, amongst others. A comprehensive review of steerable bone drills is essential due to the significant potential these devices hold for advancing minimally invasive surgical techniques and improving patient outcomes. Both scientific literature, presenting the current possibilities and patent literature highlighting future developments, are included in this review. The goal is to provide a comprehensive insight into the current state of the art and potential developments in steerable bone drill technologies. By doing so, we highlight innovative design strategies, evaluate their clinical potential, and identify areas for future development. This information is crucial for guiding further research, informing clinical practices, and ultimately enhancing the safety and effectiveness of orthopedic surgeries.

## 2 Method

### 2.1 Search method

The identification of relevant literature commenced with a search in the Scopus database to identify scientific literature providing insights into the current possibilities in steerable bone drilling. Additionally, a literature search was conducted in the Espacenet database to identify patent literature illustrating future developments in the field of steerable bone drills.

The scientific literature search performed in the Scopus database facilitated Boolean search queries. The search query in Scopus was categorized into three sections: (1) Application area (bone, osteo*, orthop*), (2) Instrument type (drill*, burr*, ream*, trep*, bore), and (3) Steering functionality (flex*, steer*, maneuv*, manouv*, deflect*, curv*, articulat*, directional*, orient*, deviat*, bend). We did not apply a date range or document type filter to our search query to ensure we encompassed all relevant studies concerning steerable bone drills. The search was, however, limited to English literature using the ‘LIMIT-TO’ function. The search query used was as follows:

TITLE (drill* OR burr* OR ream* OR trep* OR bore) AND TITLE-ABS ((bone OR osteo* OR ortop*) AND (flex* OR steer* OR maneuv* OR manoeuv* OR deflect* OR curv* OR articulat* OR directional* OR orient* OR deviat* OR bend*)) AND (LIMIT-TO (LANGUAGE, “English”))

The patent literature search was conducted in the Espacenet database, which allows for Boolean searches combined with classification searches. A Boolean search was performed within the class “A61B17/16”, that includes surgical instruments, devices or methods for bone cutting, breaking or removal means other than saws. The Boolean search was performed to find patent literature with the correct instrument type (drill*, burr*, ream*, trep*, bore) and steering functionality (flex*, steer*, maneuv*, manouv*, deflect*, curv*, articulat*, directional*, orient*, deviat*, bend), as the application area was already covered by the classification search. This resulted in the following search query:

cpc = “A61B17/16/low” AND ((ta = “drill*” OR ta = “burr*” OR ta = “ream*” OR ta = “trep*” OR ta = “bore”) AND (ta = “flex*” OR ta = “steer*” OR ta = “maneuv*” OR ta = “manoeuv*” OR ta = “deflect*” OR ta = “curv*” OR ta = “articulat*” OR ta = “directional*” OR ta = “orient*” OR ta = “deviat*” OR ta = “bend*”))

Only WO (World) patent applications in English were considered, applying filter options in the Espacenet interface.

### 2.2 Included literature

The scientific and patent literature identified underwent screening to determine eligibility based on pre-set criteria. As this study aims to present an overview of steerable bone drills it was decided to only include scientific and patent literature specifically outlining the design of an instrument capable of creating a curved tunnel through bone. Literature records solely concentrating on aspects such as instrument handle design, path planning, or tissue interaction forces were excluded from consideration. Initially, the title and abstract of each record were screened to assess eligibility. For papers that could not be excluded based on title and abstract alone, the full text was screened for eligibility.

## 3 Results

### 3.1 Identified steerable bone drills

The search in the Scopus and Espacenet database resulted in 337 identified articles and 339 identified patents (September 2023), respectively. After exclusion of inaccessible records and duplicates, and evaluation based on the eligibility criteria, 19 references from the scientific literature and 59 references from the patent literature were deemed eligible, resulting in a total of 78 included records in this study.

### 3.2 Classification steerable bone drills

**Figure 2:**
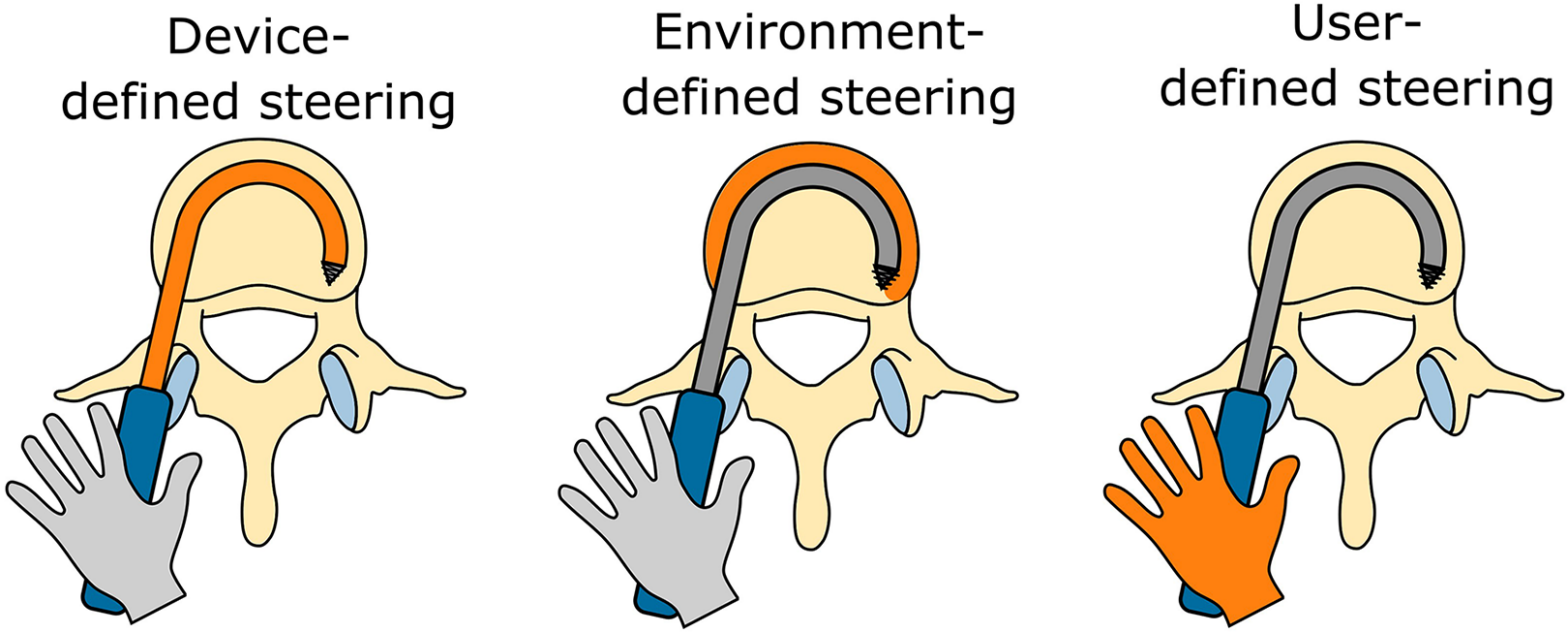
Overview of the three identified steering methods utilized by steerable bone drills: (1) device-defined, (2) environment-defined, and (3) user-defined steering.

Based on the identified scientific literature and patents on steerable bone drills, a comprehensive classification was defined based on the method of steering employed, namely: (1) Device-defined, (2) Environment-defined, and (3) User-defined steering (see Figure 2). Device-defined steering utilizes inherent mechanisms within the drill itself to control the drill path, often involving curved drills or integrated guides that determine the drill's path according to pre-programmed trajectories. Environment-defined steering relies on external factors, such as the interaction of the drill with bone density variations or other environmental cues, which guide the drill's trajectory through feedback mechanisms that adapt to the surrounding anatomy. Finally, user-defined steering places the control of the drill path in the hands of the operator, allowing for manual adjustment of the drill's direction based on real-time observations and decisions made during the procedure. In the upcoming sections, the identified drills per category will be discussed in detail.

### 3.3 Device-defined steering

Forty-seven (47) references describe bone drills in which the device primarily determines the path drilled (15–61). The instrument itself defines the pathway, either without (six references) or with a guide (41 references), shaping the curve of the drilled tunnel.

**Figure 3:**
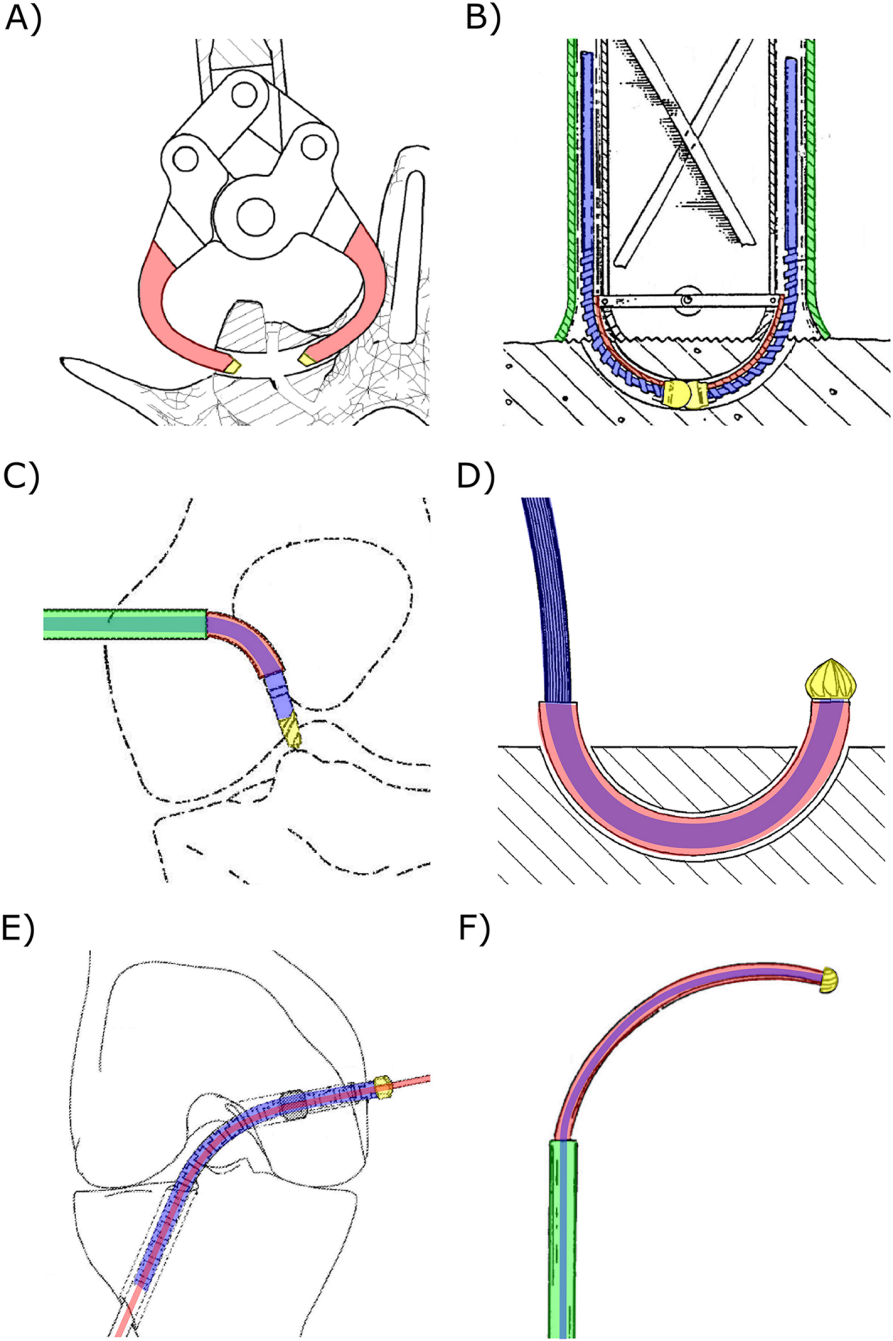
Steerable bone drills in which the drilling trajectory is defined by the device comprising a drill bit (yellow), a drive shaft (blue), an outer shaft (green) and a means to steer the drill bit (red). **(A)** Drill comprising two curved arms (red) with a drill tip (yellow) to create a curved tunnel. Illustration adapted from (15) licensed under CC BY 4.0, https://patentscope.wipo.int/search/en/detail.jsf?docId=WO2012024162&_cid=P11-M8N9FZ-94086-1. **(B)** Drill comprising two individual drill bits to create a curved tunnel. Illustration adapted from (21) licensed under CC BY 4.0, https://patentscope.wipo.int/search/en/detail.jsf?docId=WO1991011961&_cid=P11-M8NA9I-18892-1. **(C)** Drill with an external guide (red) that bends the drill bit, actuated with a flexible drive shaft (blue), to reach a desired entry point. Illustration adapted from (36) licensed under CC BY 4.0, https://patentscope.wipo.int/search/en/detail.jsf?docId=WO2018058126&_cid=P11-M8NAAU-19901-1. **(D)** Drill using an external guide (red) to create curved tunnels. Illustration adapted from (41) licensed under CC BY 4.0, https://patentscope.wipo.int/search/en/detail.jsf?docId=WO2008031245&_cid=P11-M8NA58-15412-1. **(E)** Drill using an internal guide wire (red) over which the tubular flexible drill (blue and yellow) is advanced. Illustration adapted from (44) licensed under CC BY 4.0, https://patentscope.wipo.int/search/en/detail.jsf?docId=WO2019161436&_cid=P11-M8NA3E-14038-1. **(F)** Drill comprising two concentric tubes that can be rotated and translated with respect to each other such that the curved innertube (red) can be used to steer the drill. Illustration adapted from (57) licensed under CC BY 4.0, https://patentscope.wipo.int/search/en/detail.jsf?docId=WO2001060262&_cid=P11-M8NA0W-11964-1.

In six references (15–20), the insertion of a curved drill to create a curved tunnel in bone is presented. These drills deviate from conventional axial rotation for bone removal, and instead rely solely on a pushing force to advance the curved drill into the bone, thus creating the desired curved tunnel. For instance, Blain and Kovach (15) propose a tool that allows the surgeon to advance two curved arms with a flat, beveled, or stepped tip into the bone to create a circular tunnel (Figure 3A). Similar rigid curved members are proposed to increase the fixation strength of bone anchors (16). Sohn (17) introduces a curved needle designed for insertion into the bone to create a curved tunnel for suture placement. The needle, constructed from an elastic material like nitinol, possesses the capability to be straightened using a guide prior to ejection, facilitating access to the suture site. Upon ejection, the needle disengages from the guide and reverts to its original curved shape, effectively creating the desired curve. Orbay et al. (18) outline a similar principle for fixing bone fractures using curved nails and Brockman and Vangemert (19) utilize the concept to create a curved void for spinal decompression. In contrast, Coope (20) introduces a elastic but straight nail with only an angulated tip, which results in the creation of a curved path due to the resultant forces of the tip and the angulation of the tip when hammering the nail in the bone.

An axially rotating drill, utilizing a rigid guide, can create a tunnel along a pre-defined curve (21–24). These types of drills consist of a drill tip actuated with a flexible drive shaft, loosely connected to the rigid guide. Advancing the drill with the curved guide into the bone results in the drilling of a curved tunnel. An example of a drill utilizing this principle uses disposable cartridges, each including two individual drill bits (Ø2 mm) working together to form one curved tunnel with a diameter of approximately 8 mm (Figure 3B) (24). The drill tip operates at a speed up to 700 rpm, and each cartridge is capable of drilling three to five tunnels (23, 24).

The use of an external guide in combination with a flexible drill allows the surgeon to access certain entry points without risking damage to surrounding tissue, as the guide steers the drill in the desired direction while shielding the surrounding tissue from the rotating drill bit (25–43). Saw et al. *(*36) describe a drill design comprising a drill bit actuated by a drive shaft with increased flexibility due to laser-cut slots (Figure 3C). The driveshaft is fed through a curved external guide, ensuring the drill tip is oriented in the desired direction to reach the entry point. A serrated edge on the distal end of the guide can be added to engage with the bone, preventing slipping of the drill tip (31, 32). Besides curving the drill to reach the desired entry points and shielding the surrounding tissue from damage, an external guide can also be used to create a curved tunnel in bone (Figure 3D) (37–41). Siegal et al. (42) propose the use of a segmented guide that, once extended from the rigid and straight outer sleeve, curves in a pre-determined manner based on the tensioning element and the shape of the segments that may interlock. This way, the flexible driveshaft and drill tip is oriented to drill a curved tunnel. A flexible drive shaft used in combination with an external guide can be achieved by employing a flexible material such as nitinol (30, 31, 33), incorporating (laser) cut notches or a spiral pattern (27, 31, 36), using interlocking rigid segments (31), implementing a reduction in diameter (28, 30, 32) or employing a drive cable (29, 34, 35, 38, 40, 41).

Alternatively, instead of using an external guide, an internal guide can be employed to direct the drilling trajectory of a bone drill (44–52, 61). These drills comprise a canulated drill bit actuated with a flexible driveshaft featuring a central lumen. The drill can be advanced over a pre-placed guide that defines the drilling trajectory. The drill design proposed by Walker (44) introduces a drill tip (≥Ø4.5 mm) and a flexible drive shaft with a central lumen, enabling advancement of the drill over a pre-placed guide wire (Figure 3E). The flexibility of the drive shaft is achieved through the use of a flexible material (49), hinged rigid parts, a hollow torsion cable (46, 47), or the use of interlocking segments (44, 45, 48, 50, 51). Billon et al. (51) also propose a cannulated drill that can be advanced over an internal guide. However, in this design a set of guide pins with varying radii is introduced, allowing the surgeon to choose the appropriate guide to arrive at the desired location. For drills that curve due to the use of a guide, the friction between the flexible drive shaft and a rigid outer tube can be minimized by incorporating a low-friction coating such as PolyTetraFluoroEthylene (PTFE) (51) or electroplating nickel to create a precise finish (53).

Lv et al. (54) propose using a nitinol guide over which the flexible canulated drill can be advanced. The super-elastic nitinol internal guide allows drilling straight tunnels by retracting the drill with the internal guide within the straight rigid sleeve. After drilling the straight tunnel, the internal guide can be advanced to drill a curved tunnel. Teitelbaum et al. (55) describe using two concentric guides through which a flexible drive shaft runs, actuating the drill bit at the distal end. The outer guide is rigid and straight while the second tubular guide is made of a more flexible material and has a curvature. This drill can create straight holes when the second guide is completely withdrawn within the straight outer guide. After reaching the desired length of the straight tunnel, the flexible drill with the flexible guide can be advanced to drill a circular arch (Figure 3F). A similar working principle is described in the patents by Amadio et al. (56) and Cragg and Kegan (57) and scientific papers (58–60). For example, Sharma et al. (59) propose using a flexible torque coil to actuate the drill tip, a curved nitinol guide, and a surrounding rigid stainless steel guide. The achieved radius of the drill was 35.7 mm with a Ø6.7 mm drill bit. Deviations in the drill path caused by deformations of the nitinol guide due to the interaction forces with the bone could be decreased by increasing the rotational speed of the drill tip and decreasing the insertion speed of the drill (59).

### 3.4 Environment-defined steering

Twelve (12) bone drills create a curved pathway, where the interaction with the environment is the major determinant of the drilled path (20, 62–72).

The interaction forces with the bone can be used to deflect the drill and influence the drilling trajectory as proposed by Tornier et al. (63). The proposed drill consists of a single tube with a flexible section created by a cut spiral pattern with interlocking teeth. These teeth allow for the transmission of the oscillatory actuation of the drill tip, and the shape of the teeth also defines the radius of curvature of the drill.

**Figure 4:**
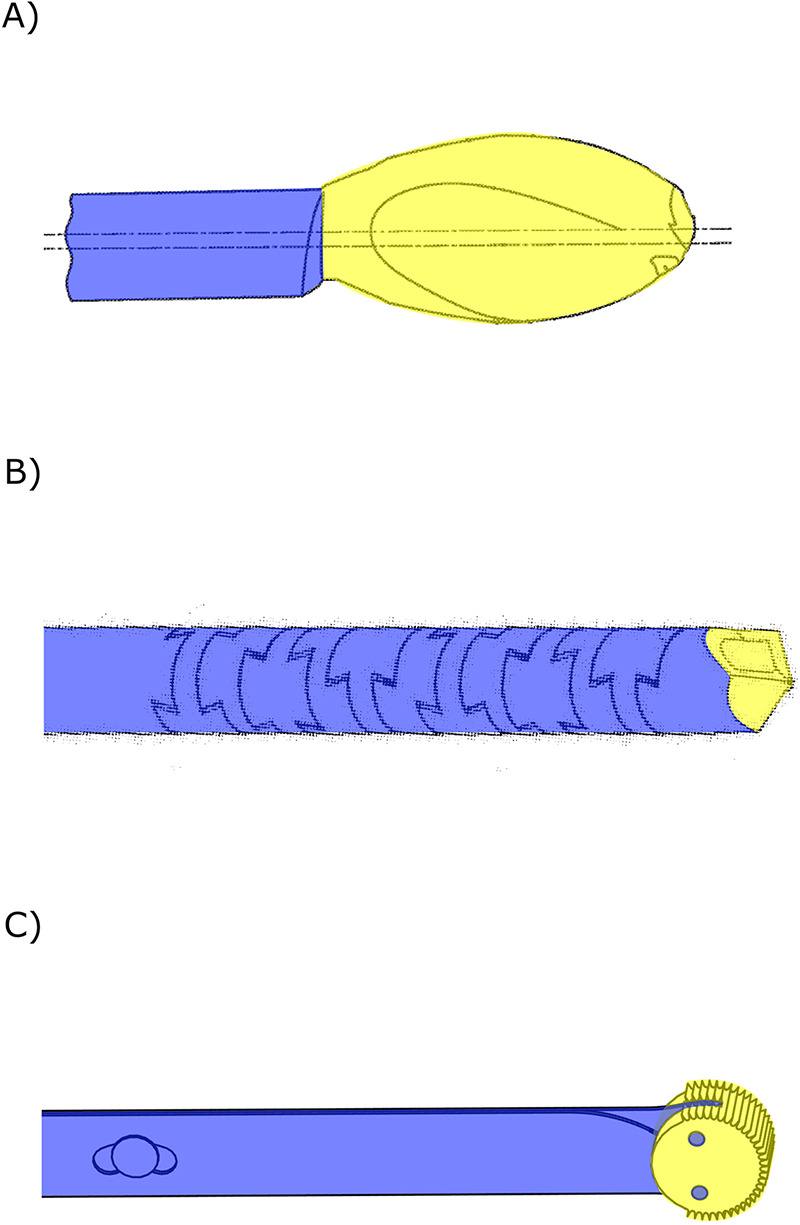
Steerable bone drills in which the drilling trajectory is defined by the environment comprising a drill bit (yellow) and a flexible drive shaft (blue). **(A)** Bone drill with an eccentric drill bit aiding bending of the drill. Illustration adapted from (64) licensed under CC BY 4.0, https://patentscope.wipo.int/search/en/detail.jsf?docId=WO2015069675&_cid=P11-M8NADG-22192-1. **(B)** Bone drill with a flexible drill shaft (blue) comprising interlocking teeth. Illustration adapted from (68) licensed under CC BY 4.0, https://patentscope.wipo.int/search/en/detail.jsf?docId=WO2021050046&_cid=P11-M8NAO5-30611-1. **(C)** Drill comprising two stacked leaf springs (blue) that allow for planar bending of the drill bit (yellow). Illustration based on bone drill developed by de Kater et al. (71).

Other references describe the utilization of the difference in resistance between the compact cortical outer layer and the softer porous cancellous bone on the inside of bone to steer the bone drill (64–72). These variations in tissue interaction forces cause the drill to deflect, taking the path of the least resistance and creating a curved tunnel. These bone drills include flexible drive shafts that actuate the drill tip and steers passively based on the tissue interaction forces. The design by McManus (64) employs an eccentric drill tip connection to aid the deflection of the drill to create a tunnel along the cortical wall for improved intermedullary nail fixation (Figure 4A). The flexible drill by Ohashi et al. (65) is designed for bone graft harvesting and comprises a flexible stainless-steel rod to transmit a rotating motion to the drill tip. The drill includes a flexible outer canula with an outer diameter of Ø3.5 mm and a length of 250 mm through which the graft material is harvested (65–67). The drill design described by Papenfuss (68) is also intended for bone marrow collection and features a drive shaft used to actuate the drill tip at the distal end while allowing for bone marrow collection through the lumen (Figure 4B). The flexible hollow drive shaft consists of rigid interlocking segments (68, 69, 72).

Where all environment-defined steering drills described thus far utilize an axially rotating motion of the drill bit, the drill by de Kater et al. (71) employs an oscillating rotation perpendicular to the drilling direction using and abrasive wheel (Figure 4C). This unique drill tip is actuated by two stacked leaf springs, allowing the drill to deflect in a single plane and follow the cortical bone layer.

### 3.5 User-defined steering

Twenty-three (23) bone drills create a curved tunnel in which the user is the major determinant of the drilling trajectory (36, 61, 72–92). The user can actively articulate the drill tip, enabling the user to steer the drill along the desired trajectory during the procedure. This steering motion is often facilitated by cables running through the drill, attached at the distal end of the drill. Pulling on one of these cables introduces a specific direction of bending in the drill, giving the user control over the drilling trajectory.

**Figure 5:**
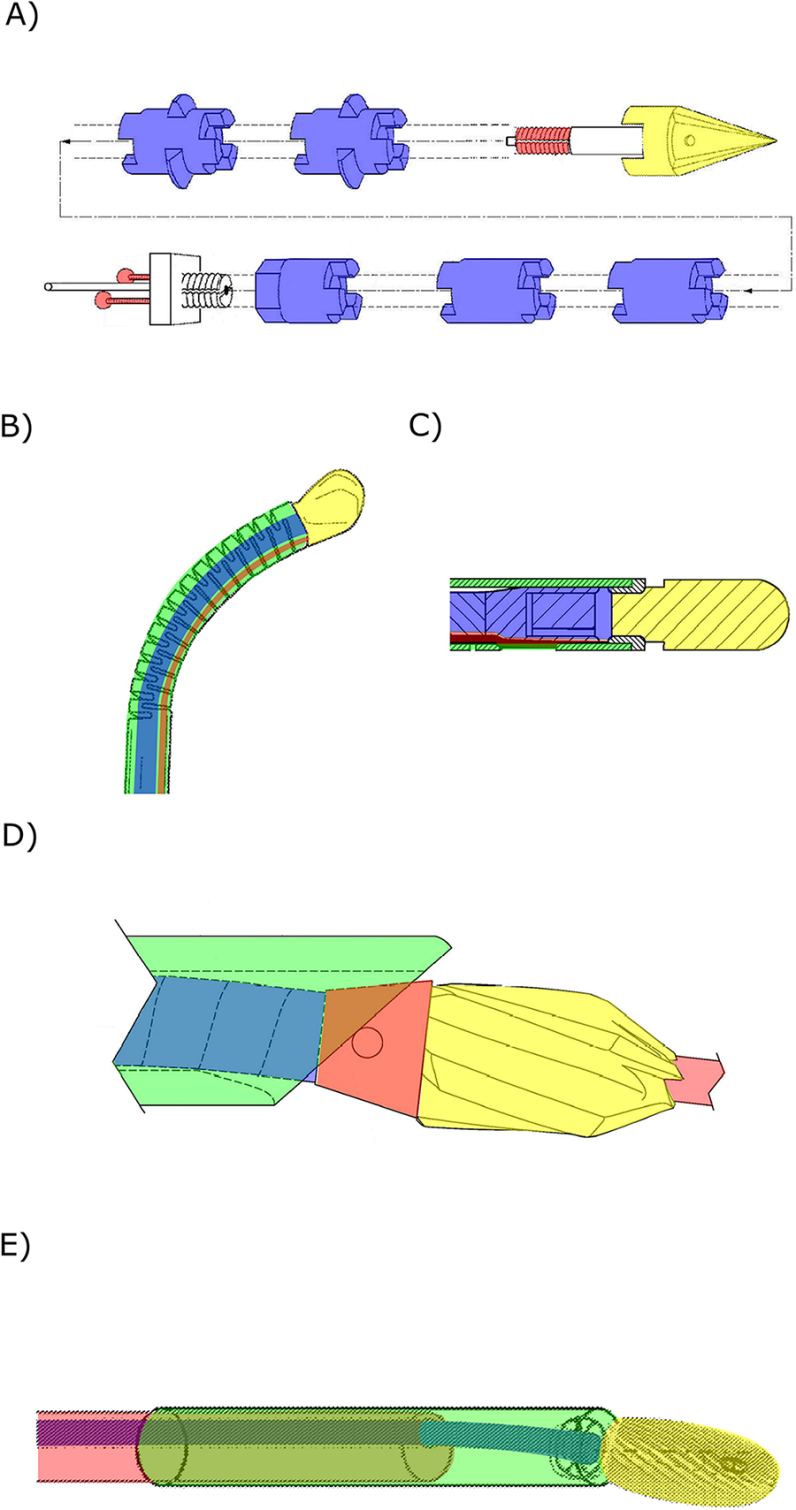
Steerable bone drills in which the drilling trajectory is defined by the user comprising a drill bit (yellow), a drive shaft (blue), an outer shaft (green) and a means of steering (red). **(A)** Steerable bone drill comprising two spines that allow articulation of the drill bit to steer the drill in the desired drilling trajectory. Illustration adapted from (72) licensed under CC BY 4.0, https://patentscope.wipo.int/search/en/detail.jsf?docId=WO2014185887&_cid=P11-M8NB78-47390-1. **(B)** Steerable drill using a steering cable (red) that can be pulled to steer the drill in the desired direction. Illustration adapted from (79) licensed under CC BY 4.0, https://patentscope.wipo.int/search/en/detail.jsf?docId=WO2018160269&_cid=P11-M8NB80-48077-1. **(C)** Drill with a drill tip comprising a helical spring-like outer shaft (green) that can be bend by tensioning the steering cable (red). Illustration adapted from (87) licensed under CC BY 4.0, https://patentscope.wipo.int/search/en/detail.jsf?docId=WO2010135606&_cid=P11-M8NB8X-48777-1. **(D)** Bone drill using an internal guide (red) as well as a drive shaft (blue) with a conical end (red) that can be tensioned to change the drill tip orientation. Illustration adapted from (61) licensed under CC BY 4.0, https://patentscope.wipo.int/search/en/detail.jsf?docId=WO2018075925&_cid=P11-M8NBAW-50313-1. **(E)** Steerable bone drill comprising two concentric flexible tubes that can be translated and rotated with respect to each other to articulate the drill tip through the eccentric drive shaft (blue). Illustration adapted from (89) licensed under CC BY 4.0, https://patentscope.wipo.int/search/en/detail.jsf?docId=WO2023023634&_cid=P11-M8NBBN-50812-1.

Five found drill designs (72–76) consist of a discrete number of rigid segments connected by joints that can be bent by the user. The drill presented by Wang et al. (73) has a tip segment that is able to make sharp curves due to the geared rolling joint connecting the segment to the rigid shaft (Ø4.5 mm). The articulation of the drill tip is achieved by steering wires, and the drill tip is actuated with a U-joint transmission through the central lumen of the drill. Similar designs are proposed in the scientific literature, comprising three jointed segments that can be articulated with steering wires, allowing the drill to drill curved tunnels (74, 75). The drill tip is actuated by a flexible drive shaft located in the center of the segments, allowing a rotational speed of up to 3,000 rpm. The drill bit can also be actuated by a sleeve of interlocking segments as presented by Bromer (72). This drill design can drill curved tunnels based on the interaction forces with the surrounding bone tissue, but the drill tip can also be articulated by the user by either tensioning or loosening one of the two spines that runs through the center of the drill, causing the drill tip to deflect (Figure 5A). Ten drill designs (36, 77–85, 92) describe the use of a central flexible drive shaft for actuation of the drill tip. This drive shaft is surrounded by a notched cannula which establishes a flexible outer sleeve due to compliant hinges. The advantage is that these compliant hinges are easier to manufacture at a small scale compared to regular joints, making miniaturization of these drills more feasible (Figure 5B). The drill of Solzbacher et al. (77) comprises a notched outer sleeve (Ø8 mm) of nitinol, allowing the drill to make a 90^o^ curve in one plane while generating high stiffness in the perpendicular plane. Steering cables run through the outer sleeve and are connected to the distal end to bend the drill bit in the desired direction.

An alternative to the notched outer sleeve is a helical spring-like structure that can be used to create a flexible outer sleeve (86–88) (Figure 5C). Watanabe et al. (86) propose the use of an outer sleeve in a drill design that comprises a number of springs. Deflection of the drill tip is aided by incorporating springs with a lower stiffness closer to the drill tip, while the springs at the base of the drill are stiffer to ensure that input via the steering cables have a minimal effect on the drill shaft.

Steering the drill by changing the drill tip configuration or orientation is also proposed to allow the user to influence the drilling trajectory (61, 89, 90). The drill design presented by Voor et al. (61) comprises a flexible outer shaft with a beveled end through which the drive shaft for the drill bit with a conical end runs (Figure 5D). Pulling the drill tip in the flexible shaft results in a change in the orientation of the drill tip and thus a change in the drilling trajectory. The drill design presented by Alambeigi and Liu (89) proposes an alternative drill design that comprises two flexible concentric tubes, of which the inner tube has an eccentric hole through which the flexible drive shaft runs (Figure 5E). Translating and rotating the inner tube relative to the outer tube changes the exit angle of the drill bit, allowing the user to change the drilling trajectory. A similar principle is proposed by Siccardi et al. (90).

Ju (91) presents a drill that does not use axial rotation but employs an electrode to remove material surrounding the drill bit by bringing the tissue in a gaseous state through the creation of a high energy electric field. This design minimizes the forces acting on the drill, allowing the use of a catheter tube containing the electrodes and steering cables to influence the drilling trajectory.

## 4 Discussion

### 4.1 Comparative analysis

The objective of this study was to provide a comprehensive overview of steerable bone drills as documented in both scientific and patent literature. The steering methods utilized by these bone drills were categorized in three groups: (1) device-defined, (2) environment-defined and (3) user-defined steering. Notably, more than half (57%) of the steerable drill designs utilizes a steering method in which the device plays a major role in determining the drill path, resulting in predefined drill paths.

The majority of drill designs discussed in this review originate from patent literature (76%). While these designs exhibit potential, a validation via proof-of-principle experiments is not required before patent publication. Consequently, the feasibility of these drill designs in a clinical setting remains uncertain. Even with the proof-of-principle experiments found in the scientific literature, not all critical aspects are thoroughly explored. For instance, prolonged heat generation during drilling can lead to bone necrosis (93), but is not specifically explored in the drill designs presented in the included literature, leaving these effects unknown.

The three primary steering methods for steerable bone drills — device-defined, environment-defined, and user-defined steering — each come with distinct advantages and disadvantages that influence their clinical application. Device-defined steering offers predefined drill paths through integrated mechanisms, inhibiting active adjustment of the drill path, but operating at increased precision over environment- and user-defined steering methods. As of now, device-defined steering drills are the only commercially available option and include examples like the *Carevature Dreal* (12), the *Stryker MicroFX OCD* (11), the *Lenkbar FlexMetric®* (94), the *Zimmer Biomet Precision Flexible Reaming system* (95) and the *DePuy Synthes Cavity Creation Instrument* (96). In contrast, environment-defined steering adapts to changes in the material properties of the surrounding bone, offering a more responsive and adaptive approach. This method, however, relies on passive steering mechanisms sensitive to individual variations in bone density, posing safety concerns due to unpredictable drill paths. Despite their straightforward design and potential advantages, these drills have not yet reached commercial markets, possibly due to concerns about the risk of cortical bone breach, especially in patients with conditions like osteoporosis. User-defined steering provides surgeons with direct control over the drill's trajectory, allowing for immediate adjustments and flexibility during procedures. While this method can enhance surgical precision, it is more prone to user error and requires complex, user-friendly interfaces to provide precise information about the drill tip's location and orientation. The need for advanced navigation systems may explain why user-defined drills remain in the pre-clinical phase. Each steering method presents a unique balance of control, adaptability, and reliance on either technology or user skill. To address these challenges, the development of hybrid drilling systems that incorporate elements from all three steering methods—combining device-defined precision, environment-defined adaptability, and user-defined flexibility—could provide greater efficacy and safety in complex surgical scenarios, paving the way for future advancements in orthopedic surgery.

**Figure 6:**
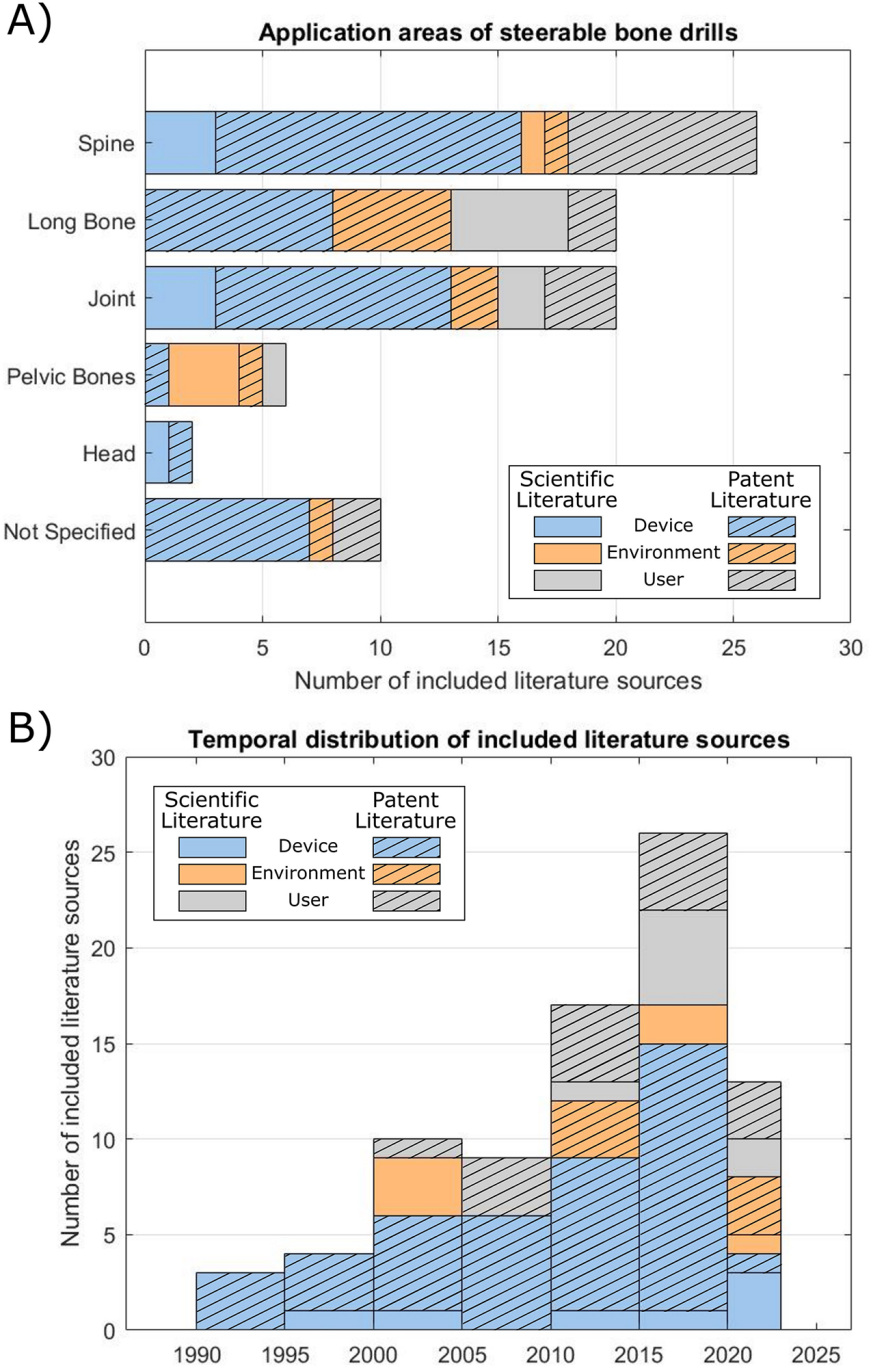
Overview of the included literature. **(A)** Application area of the steerable bone drills described in the included scientific and patent literature. **(B)** Temporal distribution of the publication date of the included literature.

The primary application areas of the drills presented in the included literature is for spine surgery procedures (33%), see Figure 6A. These procedures range from fracture fixation and vertebra decompression to reaching specific target sites such as the intervertebral disk, lesion areas or tumors. Another substantial application area involves steerable drilling through joints. In this application field, the scientific and patent literature records propose steerable bone drills for ligament reconstructions, arthroplasty to restore the function of a joint by resurfacing the bone, or placement of an artificial joint. Ten records (13%), all of which are patents, do not clearly specify an application area for the proposed steerable drill design.

From the temporal distribution of the included literature, it becomes evident that the development of steerable bone drills is relatively recent, arising from 1990 onwards, see Figure 6B. Since then, the number of steerable bone drills described in literature has been steadily increasing over the years. The development of drills that use a user-defined steering method is more recent, with literature published from 2000 onwards. This trend may be attributed to the need for a user-friendly interface to steer the drill in the desired direction, which may increase the design complexity. Furthermore, effective use of a user-defined steerable bone drill demands real-time and accurate information of the drill's location allowing the user to steer the drill in the desired path to avoid damage to the surrounding tissue. While fluoroscopy is currently employed in spine surgery for real-time 2D navigation, this method exposes both the patient and clinicians to radiation (97). Alternative navigation possibilities, such as Diffuse Reflectance Spectroscopy (DRS), could be explored in order to real-time detect cortical breaches to prevent damage to surrounding anatomy (98).

### 4.2 Limitations and future research

This review offers a comprehensive overview of steerable bone drills described in both patent and scientific literature. The majority of the drills included in this review employ a conventional axial rotating drill bit. For future research, exploring alternative drilling methods and their implications on steerable bone drill development would be insightful. Alternative drilling methods, such as water jet drilling or piezoelectric drilling, are associated with higher quality of the cuts and less thermal damage to the surrounding bone (99, 100). Furthermore, waterjet drilling is presented as an alternative drilling method that could potentially ease the development of a steerable bone drill by using flexible tubing (99). However, these alternative drilling methods have not been utilized in the included steerable bone drills, making them an interesting topic for future research.

The broader clinical adoption of steerable bone drills faces several technological challenges that must be addressed to enhance their effectiveness and integration into surgical practices, see also Table 1. One primary limitation is the precision and reliability of current steering mechanisms. Although, the included literature presents various validation experiments of the proposed designs, these validation experiments were generally limited to phantom studies, which usually entail homogenous materials, indicating that there are still steps to be taken before steerable bone drills can be applied in a clinical setting.

**Table 1:** Comparison of steering methods of steerable bone drills.

Steering method	Device-defined steering	Environment-defined steering	User-defined steering
Versatility	Low versatility, due to predetermined drill path defined by the device. Path adjustments impossible during the procedure.	Medium versatility, potential path adjustments based on the interaction forces with the environment and potential to drill a path along the cortical bone layer.	High versatility, adjustment to the path during the procedure possible by inputs of the user.
Precision	High precision due to predetermined drill paths using rigid guides.	Precision dependent on the bone quality and thus interaction forces.	Precision dependent on the user. Potential high precision with proper feedback systems.
Reliability	Very reliable due to the use of rigid guides.	Reliable, but failure possible when encountering excessive interaction forces.	Reliable, but failure possible due to flexible moving drill elements.
Safety	High, dependent on proper pre-operative planning.	Medium, dependent on bone quality. Potential of cortical breach with low bone quality.	Medium, dependent on the skill of the operator. Prone to user error.

Comparing the various steering methods of steerable bone drills with traditional straight-path drilling in quantitative terms reveals significant advantages in terms of surgical precision. Additionally, these advanced steering technologies have the potential to decrease the operative time by allowing more precise targeting of the lesion and improving patient outcomes. However, another factor that needs to be considered is the potential increased risk of complications due to user-error. Further research should, therefore, be executed to determine the advantages of using non-straight drill paths on the long-term success rates of orthopedic procedures.

Another aspect that is partly overlooked, but necessary to enable steerable bone drilling in a clinical setting, is the navigation of the drill. Using a steerable bone drill in clinical practice requires extensive real-time knowledge of the drill's location and the patient's anatomy. Particularly, drills intended for actively steering by the user necessitate real-time awareness of the drill's location within the patient's anatomy, allowing the surgeon to guide the drill in the preferred direction during the procedure and can be used as a means of safeguarding patient safety. Unfortunately, the integration of advanced sensors and feedback systems to improve steering precision remains a technical challenge due to the dimensional constraints. Especially in environment-defined steering integrating feedback systems is challenging, as these systems need to be highly responsive and adaptive to dynamic changes in bone morphology and density during procedures. Incorporating real-time navigation techniques for steerable bone drills would be crucial for advancing their development.

The integration of steerable bone drills with robotic systems presents a promising avenue for overcoming these challenges. Robotics can enhance the precision and repeatability of steerable drills by incorporating advanced algorithms for path planning and real-time adjustments of the drill path. Robotic systems can also integrate sophisticated imaging or shape sensing technologies, such as Computed Tomography (CT) or optical fibers containing Fiber Bragg Gratings (FBGs), to provide continuous feedback and improve the accuracy of the different steering methods. Future research should focus on developing robotic platforms that can be integrated with steerable drills and allow for easy integration into the orthopedic procedures.

## 5 Conclusion

Steerable bone drills have potential to significantly benefit a range of (orthopedic) procedures by enhancing maneuverability, facilitating access to target areas, and minimizing damage to surrounding anatomy. This study offers a comprehensive overview of steerable bone drill designs as documented in both patent and scientific literature. The search query, coupled with pre-defined inclusion and exclusion criteria, resulted in the inclusion of 59 patent and 19 scientific literature references. Based on the included references, it was found that the drilling trajectory could be defined by: (1) the device, (2) the environment, or (3) the user. In the first category, the drilling trajectory is integrated into the bone drill and is thus predetermined before the procedure. In the second category, the drilling trajectory is defined during the procedure based on the tissue interaction forces between the drill and the surrounding tissue. The drills in the third category enable the user to adapt the drilling trajectory during the procedure. This comprehensive overview aims to provide insights into the current and future development of steerable bone drills, serving as a valuable source of inspiration for the development of innovative steerable bone drill designs.
